# Inferior Parietal Lobule Encodes Visual Temporal Resolution Processes Contributing to the Critical Flicker Frequency Threshold in Humans

**DOI:** 10.1371/journal.pone.0098948

**Published:** 2014-06-06

**Authors:** Andrea Nardella, Lorenzo Rocchi, Antonella Conte, Matteo Bologna, Antonio Suppa, Alfredo Berardelli

**Affiliations:** 1 IRCCS Neuromed, Pozzilli, Isernia, Italy; 2 Department of Neurology and Psychiatry, "Sapienza", University of Rome, Rome, Italy; University Medical Center Goettingen, Germany

## Abstract

The measurement of the Critical Flicker Frequency threshold is used to study the visual temporal resolution in healthy subjects and in pathological conditions. To better understand the role played by different cortical areas in the Critical Flicker Frequency threshold perception we used continuous Theta Burst Stimulation (cTBS), an inhibitory plasticity-inducing protocol based on repetitive transcranial magnetic stimulation. The Critical Flicker Frequency threshold was measured in twelve healthy subjects before and after cTBS applied over different cortical areas in separate sessions. cTBS over the left inferior parietal lobule altered the Critical Flicker Frequency threshold, whereas cTBS over the left mediotemporal cortex, primary visual cortex and right inferior parietal lobule left the Critical Flicker Frequency threshold unchanged. No statistical difference was found when the red or blue lights were used. Our findings show that left inferior parietal lobule is causally involved in the conscious perception of Critical Flicker Frequency and that Critical Flicker Frequency threshold can be modulated by plasticity-inducing protocols.

## Introduction

Visual temporal resolution processes in humans can be evaluated by measuring the Critical Flicker Frequency threshold (CFFt)[1], [2], defined as the frequency at which a flickering light is perceived as a continuous light [3], [4]. In normal subjects, several studies have investigated the influence on CFFt of several factors including age, blood carbon dioxide, and site of retinal stimulation [5]–[10] and confirmed that CFFt is a reliable test for the investigation of visual temporal resolution [11]. Although the contribution of anterior visual pathways to the temporal analysis of visual stimuli is well established [12]–[14], the physiological basis of CFFt and in particular the role played by the cortical areas in conscious perception of a flickering light is still unclear. Several studies using intracranial or scalp recording of electrical activity in animal models [14], [15] and in humans [13], [16], [17] suggest that neural activity in primary visual cortex (V1) can be driven by the frequency of a flickering light. Activity of V1, dependent on the frequency of the visual stimulation during exposure to a flickering light, has been confirmed by studies in humans with positron emission tomography (PET) [18], [19] and functional magnetic resonance imaging (fMRI) [20]. However, it is not clear whether the activity in V1 is associated with conscious perception of a flickering light or if merely reflects early stages of visual stimuli processing. Other authors provided information on cortical areas involved in CFFt encoding by showing left inferior parietal lobule (IPL) activation [21], a cortical area known for its important role in visual awareness [22], [23]. Besides V1 and left IPL, more complex functional areas may participate in CFFt perception, including the so called mediotemporal cortex (hMT/V5+) [24]. hMT/V5+ is well known for its importance in the analysis of visual motion processing [25]–[27] but its role in CFFt perception needs further clarification. Additional information on the contribution of different cortical areas to CFFt is needed to better understand the pathophysiological mechanisms of altered temporal processing of visual stimuli reported in several neurological disorders [10], [28]–[34].

In this study we used repetitive Transcranial Magnetic Stimulation (rTMS) to investigate the role of striate and extra-striate cortical areas in the CFFt. rTMS is a non-invasive technique which induces changes in cortical activity and plasticity mechanisms outlasting the stimulation by several minutes or longer [35]. Among the different rTMS protocols, continuous Theta Burst Stimulation (cTBS) is able to induce inhibition of cortical activity through a Long Term Depression (LTD-like) plasticity mechanism with a good spatial resolution in a fast, reversible way [36]. Thus, cTBS lends itself to the investigation of the specific contribution of various cortical areas in a given behavioral task [37]–[39].

In a first series of experiments, we investigated changes in CFFt induced by cTBS over V1, IPL and left hMT/V5+. CFFt was tested with the ascending and descending method [29] and with different light wavelengths (red and blue). After finding that cTBS over the left IPL altered the CFFt, whereas cTBS over V1 and left hMT/V5+ did not, we investigated whether cTBS applied to right IPL also influenced the CFFt.

## Materials and Methods

### Subjects

Twelve healthy volunteers (7 male and 5 female, mean age 28.1±3 years) took part in the study after giving their written informed consent. All the participants were right-handed and had normal or corrected-to-normal visual acuity; none of them had ophthalmological disease and were assuming drugs active at CNS level at the time of the experiments. The experimental procedures were carried out in accordance with the Declaration of Helsinki and approved by the institutional review board of the Department of Neurology and Psychiatry, “Sapienza” University of Rome.

### Procedure for the critical flicker frequency threshold measurement

Subjects were seated in an armchair, in a dimly lit room. They were asked to look at the hole in the flicker machine with one eye, keeping the other eye closed, and report orally whether they perceived the light as flickering or continuous. The CFFt was measured by means of two light emitting diodes (LEDs), one of which was red and the other blue, with a black background (Red LED: Optosupply mod. OSER5131A-QR, 625 nm wavelength; Blue LED Microelectronics mod. MBB51DA, 430 nm wavelength). Luminance was set at 300 cd/m^2^ for both LEDs. The LEDs frequency was manually adjusted using a knob on the device and continuously monitored on a PC screen connected with the CFF device using Visual Analyser 6.0 beta software. LEDs frequency ranged from 15 to 60 HZ, with a square waveform and 50% duty-cycle oscillation, rate of decrease/increase of approximately 2 Hz/sec in 0.5 Hz steps.

The CFFt was measured for both eyes separately, using the red and blue LEDs, according to continuous ascending and descending method. In the ascending method, the LED frequency was set at 15 Hz and gradually increased, and the subject had to report when the flickering LED became continuous. In the descending method, the LED frequency was set at 60 Hz and gradually decreased, and the subject had to report when the continuous LED started to flicker [10], [30], [31]. The threshold was defined as the average of the three consecutive measurements.

### Transcranial magnetic stimulation

A Magstim Super Rapid magnetic stimulator (Magstim Company, Whitland, Wales, UK) connected to a figure-of-eight coil 90 mm in diameter was used to deliver cTBS. The cTBS paradigm consisted of three-pulse bursts at 50 Hz repeated every 200 ms for 40 sec, for a total 600 pulses [36] delivered at 80% of the active motor threshold (AMT). The AMT was the lowest intensity able to evoke a motor evoked potential (MEP) of at least 200 µV in five out of ten consecutive trials during a 20–30% maximum voluntary contraction of the first dorsal interosseous muscle (FDI). The FDI cortical hotspot was in the left hemisphere for left IPL, left hMT/V5+ and V1 and right hemisphere for right IPL. As previous studies suggested [37], [40], [41], it is adequate to use motor threshold to calibrate TMS intensity for the stimulation of non motor cortical areas.

### Electromyographic recording

EMG activity was recorded through a pair of Ag/AgCl electrodes placed over the FDI muscle in a belly-tendon fashion. The raw signal, sampled at 5 kHz with a CED 1401 A/D laboratory interface (Cambridge Electronic Design, Cambridge, UK), was amplified and filtered (bandwidth 20 Hz-1 kHz) with a Digitimer D 360 (Digitimer Ltd., Welwyn Garden City, Hertfordshire, UK). Data were stored on a laboratory computer for on-line visual display and further off-line analysis (Signal software, Cambridge Electronic Design, Cambridge, UK). To ensure complete relaxation of the target muscle throughout the experimental sessions, we continuously monitored EMG activity by means of audio and visual feedback.

### Localization of cortical area of interest

cTBS was delivered over the scalp site corresponding to the left IPL, left hMT/V5+, V1 and right IPL. To localize the cortical areas of interest, we used a Polaris Vicra optical measurement system (Northern Digital Inc.) combined with the SofTaxic evolution navigator system (E.M.S., Bologna, Italy). The SofTaxic navigator system computes an estimated volume of the subject's MRI brain and guides the TMS coil position and orientation for the stimulation of the cortical hot-spot defined by the Talairach coordinates [42]. Previous studies demonstrated that the mean accuracy of the estimated MRIs is comparable to the spatial resolution of TMS [43], [44]. IPL and hMT/V5+ were localized according to the following Talairach coordinates: left IPL: (x, y, z) = −57, −30, +39; right IPL: (x, y, z) = +57, −30, +39) [21]; left hMT/V5+: (x, y, z) = −47, −72, +6) [25]. Localization of V1 corresponded to the optimal site for eliciting static phosphenes (see next paragraph).

### Phosphene threshold and moving phosphene threshold determination

The optimal site to elicit phosphenes was found positioning the coil 2 cm dorsal from the inion, with the handle pointing upwards, and moving it slightly to find the region where the brighter phosphenes could be elicited with an intensity of 80% MSO of a biphasic stimulator [45], [46], using a paired-pulses TMS paradigm (20 msec interstimulus interval) [47], [48], with 5 second-intervals between trials. The phosphenes threshold (PT) was calculated starting with an intensity of 80% MSO and decreasing by 5% until phosphenes were no longer perceived. Then, MSO was increased again in 2% steps until the minimum intensity at which the subject could perceive a stable phosphene in at least three cases out of five stimuli was established [49]. The same procedure was used to calculate the moving phosphenes threshold (MPT), except that the coil was directly placed over left hMT/V5+ (Talairach Coordinates (x, y, z) = −47, −72, +6). Moving phosphenes were defined as visual motion sensations when phosphenes appeared in the same form and moved to the same direction in at least three out of five stimuli.

### Experimental procedure

Each subject underwent cTBS over V1, left IPL and left hMT/V5+ in three separate sessions performed at least two weeks apart. The order in which the cortical areas were stimulated was randomly chosen for the first experimental session and counterbalanced across the subjects for the other two sessions. All the subjects underwent a fourth experimental session with cTBS over the right IPL. In each experimental session, the CFFt was measured at three time points: before cTBS, 5 and 30 minutes thereafter. At each measurement time point, the examiner combined the colour of LED (blue, red), the eye (left, right) and the method of CFFt measurement (ascending, descending) in a random sequence, for a total of eight measurements. The duration of the CFFt measurement at each time point took slightly less than 10 minutes. The investigator who performed CFFt measurements was blind to the TBS session. For the experimental session of cTBS over V1 and left hMT/V5+ we also measured PT and MPT respectively, before and within 4 minutes after cTBS to evaluate the efficacy of the stimulation protocol.

### Statistical analysis

Four separate three-way repeated measures ANOVAs were performed to evaluate changes in CFFt, tested with red and blue lights in ascending and descending mode, with “cortical areas”: (V1, left and right IPL and left hMT/V5+), “eyes” (right and left eye) and “time” (before cTBS, 5 minutes and 30 minutes after cTBS: T0, T1, T2, respectively) as factor of analysis. A one-way repeated measure ANOVA with factor “session” was used to compare CFFt values at baseline in the four experimental sessions. Tukey's Honest significance difference was used for the post hoc analysis. Holm's correction for multiple comparisons was used to disclose false significance. Changes in PT values before and after cTBS over V1 and changes in MPT values before and after cTBS over hMT/V5+ were analyzed with a paired sample T test. P values <0.05 were considered significant. All the values are expressed as mean ± SE. Greenhouse-Geisser's correction for non sphericity was applied when needed.

## Results

### Main experiments: effects of cTBS on CFFt

**Figure 1:**
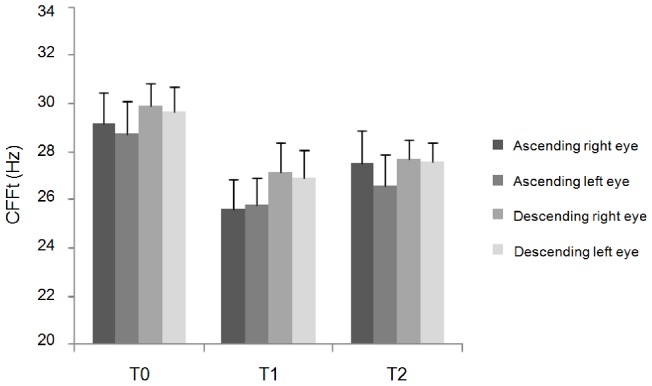
Changes in red-LED critical flicker frequency threshold induced by cTBS over left inferior parietal lobule. Changes in red LED-critical flicker frequency threshold (CFFt) measured with the ascending method and descending method on the right and left eye induced by cTBS over left inferior parietal lobule (IPL). Each column represents mean value; bars represent SE. Y axis represents CFFt values expressed in Hz. X axis represents time points (T0: before, T1: 5 minutes and T2: 30 minutes after cTBS).

**Figure 2:**
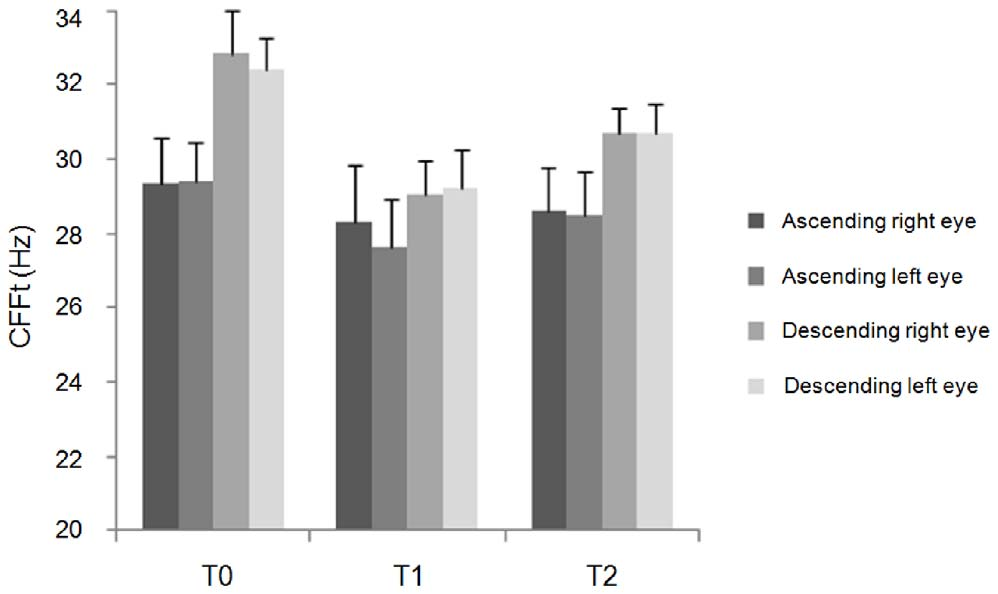
Changes in blue-LED critical flicker frequency threshold induced by cTBS over left inferior parietal lobule. Changes in blue LED-critical flicker frequency threshold (CFFt) measured with the ascending method and descending method on the right and left eye induced by cTBS over left inferior parietal lobule (IPL). Each column represents mean value; bars represent SE. Y axis represents CFFt values expressed in Hz. X axis represents time points (T0: before, T1: 5 minutes and T2: 30 minutes after cTBS).

**Figure 3:**
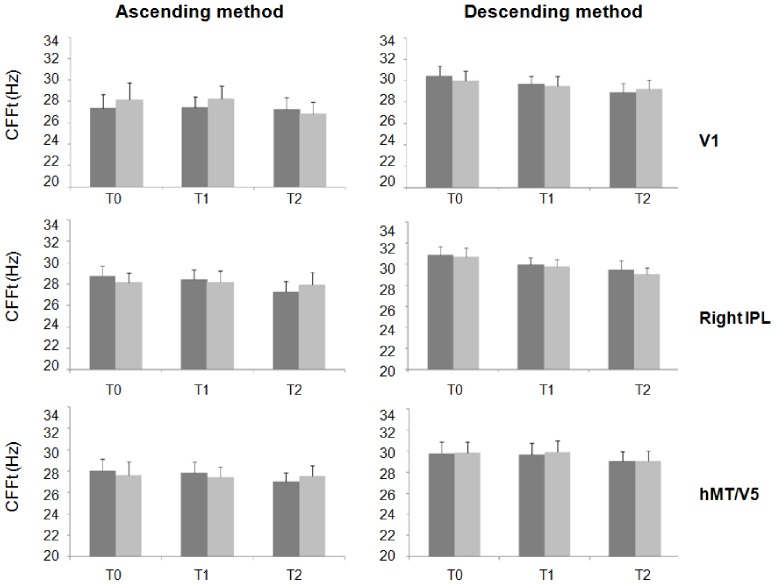
Changes in red-LED critical flicker frequency threshold induced by cTBS over right inferior parietal lobule, primary visual cortex and mediotemporal cortex. Changes in red-LED critical flicker frequency threshold (CFFt) measured with the ascending method (left panel) and descending method (right panel) in the right (dark grey column) and left (light gray column) eye induced by cTBS over right inferior parietal lobule (IPL), primary visual cortex (V1) and mediotemporal cortex (hMT/V5). Each column represents mean value; bars represent SE. Y axis represents CFFt values expressed in Hz. X axis represents time points (T0: before, T1: 5 minutes and T2: 30 minutes after cTBS).

**Figure 4:**
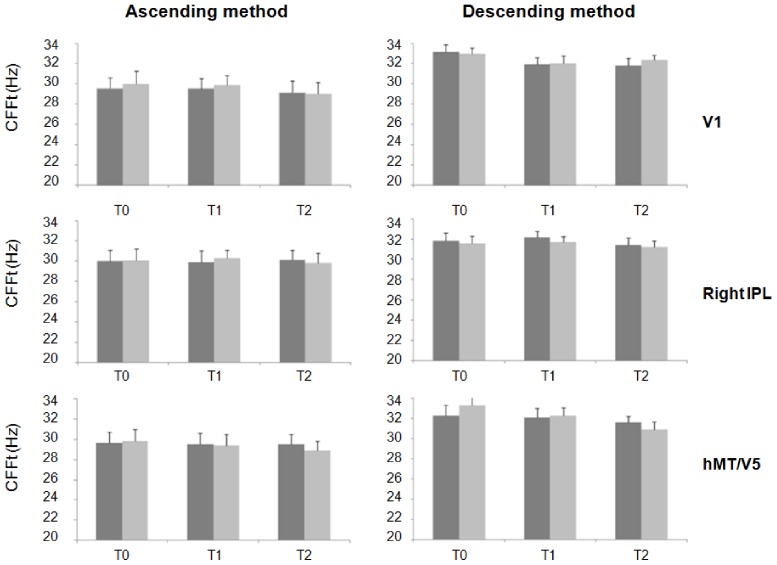
Changes in blue-LED critical flicker frequency threshold induced by cTBS over right inferior parietal lobule, primary visual cortex and mediotemporal cortex. Changes in blue-LED critical flicker frequency threshold (CFFt) measured with the ascending method (left panel) and descending method (right panel) in the right (dark grey column) and left (light gray column) eye induced by cTBS over right inferior parietal lobule (IPL), primary visual cortex (V1) and mediotemporal cortex (hMT/V5). Each column represents mean value; bars represent SE. Y axis represents CFFt values expressed in Hz. X axis represents time points (T0: before, T1: 5 minutes and T2: 30 minutes after cTBS).

Repeated measures ANOVA showed a significant interaction of factors "cortical areas" and “time” for the ascending method-red light (F(6,66) = 4.92; p<0.01), descending method-red light (F(6,66) = 2.24; p = 0.04), ascending method-blue light (F(6,66) = 2.29; p = 0.04) and descending method-blue light (F(3.01,33.1) = 3.07; p = 0.04). Post-hoc analysis showed that cTBS over left IPL significantly reduced the CFFt (Figures 1 and 2), whereas it did not when delivered over V1, left hMT/V5+ and right IPL (Figure 3 and 4). Left IPL cTBS-induced decrease in CFFt was significant at T1 and to a lesser extent at T2 for all the red and blue thresholds in both the right and left eyes (ascending method-red light right eye: T1: p<0.0005, T2: p = 0.04; ascending method-red light left eye: T1: p<0.0001, T2: p = 0.02; descending method-red light right eye: T1: p = 0.0003, T2: p = 0.02; descending method-red light left eye: T1: p = 0.001, T2: p = 0.01; ascending method-blue light right eye: T1: p = 0.01; T2: p = 0.03, ascending method-blue light left eye: T1: p = 0.002; T2: p = 0.03; descending method-blue light right eye: T1: p = 0.0001, T2: p = 0.02; descending method-blue light left eye: T1: p = 0.002, T2: p = 0.02). Repeated measures ANOVA performed to compare CFFt values at T0 in each experimental session showed that the CFFt values at T0 did not differ significantly across the experimental sessions of the ascending method-red light (F(3,33) = 2.39; p = 0.09), descending method-red light (F(3,33) = 0.48; p = 0.69), ascending method-blue light (F(3,33) = 0.07; p = 0.97) or descending method-blue light (F(3,33) = 0.92; p = 0.44) (Table 1).

**Table 1:** Critical Flicker Frequency (CFFt) and active motor threshold (AMT) values in all the experimental sessions at the baseline.

			Left IPL		V1		Right IPL		hMT/V5+	
			Right	Left	Right	Left	Right	Left	Right	Left
CFFt	Red LED Ascending method	*mean*	29.19	28.76	27.35	28.16	28.72	28.15	28.02	27.59
		*SE*	1,29	1,32	1,29	1,55	0,99	0,87	1,07	1,22
	Red LED Descending method	*mean*	29.93	29.64	30.45	29.98	30.87	30.69	29.81	29.85
		*SE*	0,70	0,88	0,90	0,84	0,79	0,82	1,02	0,99
	Blue LED Ascending method	*mean*	29.37	29.39	29.56	29.99	30.00	30.05	29.64	29.86
		*SE*	1,12	1,08	1,03	1,21	1,03	1,08	1,04	1,06
	Blue LED Descending method	*mean*	32.86	32.41	33.15	32.98	31.81	31.60	32.26	33.30
		*SE*	1,10	0,83	0,73	0,55	0,79	0,66	0,97	1,00
AMT		*mean*	40.08		39.92		40.58		40.50	
		*SE*	2,21		1,43		1,45		2,02	

In the upper panel values of Critical Flicker Frequency (CFFt), expressed in Hz, measured at baseline (T0 =  before cTBS) with ascending and descending methods red- and blue-LED stimulation for left and right eye. In the lower panel values of active motor threshold, expressed in percentage of maximum stimulator output.

### Control experiments: effects of cTBS on phosphene threshold and moving phosphene threshold

**Figure 5:**
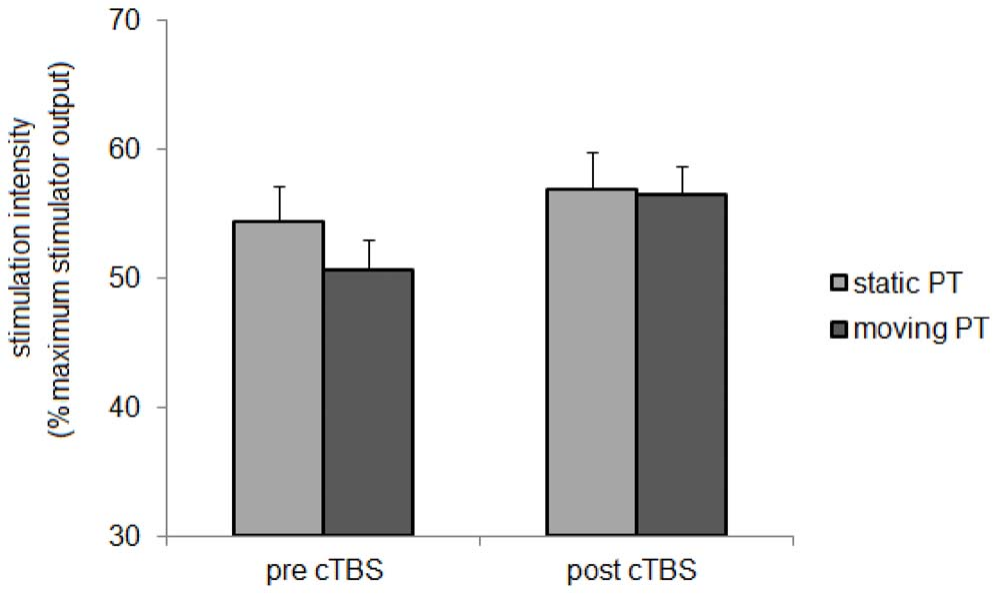
Changes in static and moving phosphenes thresholds induced by cTBS over occipital and mediotemporal cortex. Changes in static (light gray column) and moving (dark gray column) phosphenes thresholds (PT) induced by cTBS over occipital cortex and mediotemporal cortex, respectively. Each column represents mean value; bars represent SE. Y axis represents stimulation intensity expressed as percentage of maximum stimulator output. X axis represents time points (before and after cTBS)

Paired sample T test showed that cTBS delivered over V1 significantly increased PT (mean PT pre*-*cTBS = 53.5 vs. mean PT post-cTBS = 56.2; p = 0.006). cTBS over left hMT/V5+ significantly increased MPT (mean MPT pre*-*cTBS = 50.7 vs. mean PT post-cTBS = 56.6; p<0.01) (Figure 5).

## Discussion

In this study we showed that cTBS over left IPL modulated the CFFt, whereas cTBS delivered over V1, left hMT/V5+ and right IPL did not. In particular, cTBS over left IPL reduced both the ascending and descending CFFt. The CFFt test yielded similar findings for both the red and blue LEDs.

Our experimental procedures included several precautions to avoid methodological errors. During the measurement of the CFFt the eye, the colour of the LED and the method chosen (ascending and descending methods) were randomly selected to avoid bias due to the timing of examination. Moreover, randomly selecting the ascending or descending method of CFFt measurement reduced the risk of frequency adaptation in the subject (frequency adaptation can be induced by an exposure to a flickering light at the same frequency for more than two seconds) [50].

As we used a neuronavigator system, we are confident that cTBS was actually delivered over IPL and hMT/V5+. For V1 session, the use of neuronavigation allowed us to keep the coil stable during cTBS over the optimal position to evoke phosphenes. Since Thickbroom and coworkers [51] demonstrated that at low TMS intensities the current spread to neural tissue adjacent to the coil focus is minimal, the possibility that the coil we used extends the stimulation over the boundaries of the inferior parietal lobule is unlikely. Moreover, we used TMS intensities lower than those used in previous studies with TMS targeting IPL [52], [53]. Similarly to a previous study on rTMS delivered over visual cortex [37], we determined the intensity of cTBS as a percentage of the AMT, which, unlike phosphene threshold, do not rely on a subjective report. The use of M1 excitability parameters for V1 stimulation is also supported by the observation that M1 and visual cortex excitability are functionally related [40], [41]. Since the order of the three main experimental sessions was counterbalanced across subjects, we can rule out that our findings were affected by the multi-session design. Although a possible limitation of our study is that all participants underwent right IPL stimulation session as fourth session, the observation that the CFFt at baseline in all the sessions did not differ rules out a possible learning effect and also confirms that the CFFt yields reproducible data. Since the maximal stimulation frequency we used in our study was 60 Hz, we confidently exclude the possibility that our findings were affected by visual temporal resolution limits in early visual pathway. In a study using pattern-reversal steady-state visual evoked potentials and LED visual stimulation it has been demonstrated that the visual pathways from the retina to V1 can process LED visual information to an upper frequency limit of 70 Hz [54]. Using a bi-chromatic isoluminant visual stimulation the response to the chromatic flicker in the visual cortex may even occur at frequencies above that of flicker perception [55].

The changes in CFFt after cTBS over left IPL suggest that left IPL is causally involved in the conscious perception of CFF and provide further insight into the neuronal network involved in this temporal visual task. Overall, these findings are in line with fMRI data of Carmel et al. [21], who reported high cerebral activation in the left IPL during red light CFF perception. Based on previous hypothesis about temporal information processing in the central nervous system, the neural processes underlying the temporal analysis of visual information may rely on two models: a "spectral model" and a "scalar model". According to the spectral model, different time intervals are represented by the activation of non-overlapping neural elements encoding a specific stimulus frequency [56], [57]. According to the “scalar model”, also defined "population clock model", temporal codes are established through oscillatory processes which involve the activity of a network of neurons. If the hypothesis of the spectral model is valid for CFFt encoding in left IPL, cTBS should have elicited differential effects when CFFt was measured with the ascending and descending methods because the two methods imply starting the CFFt testing at different flicker frequencies. Thus, the observation that cTBS elicited similar effects on CFFt tested with the ascending and descending methods makes the hypothesis of the spectral model unlikely. Based on our results, we therefore speculate that the CFFt is encoded by the synaptic activity of a neural network in the left IPL according to a “scalar model”. Although there are few evidences on the putative cellular mechanisms underlying temporal processing, a study by Buonomano [58] suggested that timing operations in cortical circuits might rely on N-Methyl-D-Aspartate (NMDA) receptors-dependent neurotransmission. Since LTD-like plasticity relies on NMDA neurotransmission [59], [60], it is possible that left IPL cTBS-induced modulation on CFFt specifically depends on changes in NMDA-mediated synaptic activity rather than on a non-specific depression of cortical activity.

The observation that the CFFt was significantly modulated by cTBS delivered over the left, though not over the right IPL deserves a comment. Previous studies reported a lateralization of the temporal components of visual perception, as demonstrated by the larger VEP amplitude in the left hemisphere than in the right hemisphere, when the visual stimuli had a higher temporal and lower spatial resolution [61], [62]. These results point to a prevalent role of the left hemisphere in the temporal resolution of visual stimuli [21], [63]. The right hemisphere is thought to be preferentially involved in attentional processes related to visual tasks. rTMS delivered over a number of cortical areas (inferior frontal cortex, inferior temporal cortex and middle parietal cortex, respectively F8, T8 and P4 position according to the “International 10–20 System”) of the right but not the left hemisphere disturbed visual working memory [64]. In line with a prominent role of the right hemisphere in processing of discriminative aspects related to spatial resolution of visual stimuli, in an rTMS study designed to investigate the neural circuits underlying changes in visual detection, rTMS delivered over the right, though not over the left, superior parietal lobule induced “change blindness” (the inability to detect changes between two images separated by a brief time interval) [65]. Moreover, visual flicker detection was normal in patients with lesions in the right hemisphere, whereas attention-dependent visual test were altered [66]. Overall, these studies suggest that the right hemisphere is involved in visual attentional processes and in the analysis of meaningful visual stimuli, whereas the left hemisphere may be involved in the temporal analysis of visually simple stimuli such as the flickering LED used in our task.

To explain why left IPL-cTBS partially interfered with physiological processes contributing to CFFt, whereas V1-cTBS did not, we might speculate that the spatial resolution of focal cTBS might be topographically limited compared to the ample representation of visual inputs in V1 and thus not able to interfere with mechanisms underlying CFFt. However, despite leaving CFFt unchanged, cTBS applied over V1 modulated PT [47], thus showing that our stimulation protocol effectively influenced V1 activity. Occipital stimulation may involve visual areas close to V1 [67], [68], phosphenes perception, however, implies V1 activation [46], [69] and since we used phosphene threshold to determine the coil positioning, we confidently assume that we actually stimulated V1 [70]. Neurons in V1 contribute to the analysis of simple visual information like orientation and binocular disparity [71]. Although the role of V1 in visual awareness is controversial [46], [72], a number of recent studies suggest that V1 activity does not directly correlate with the subject's percept [73], and that V1 contribution to visual awareness seems limited [74]. Thus, in line with evidence from other studies [12] we speculate that V1 does not contribute significantly to the conscious perception of visual flicker.

Although we did not observe any changes in the CFFt after cTBS over left hMT/V5+, MPT increased after cTBS, demonstrating that cTBS was effective in modulating hMT/V5+ activity. The lack of changes in the CFFt after cTBS over the hMT/V5+ may be due to the functional role played by this area [26], [75]. The hMT/V5+ is a “relay” structure in the dorsal visual pathway which might be involved in movement perception more than in timing processing of visual stimuli, as suggested by several studies using single pulse TMS [25], [76], [77] and repetitive TMS protocols [27].

A limitation of the study might be that we did not investigate the effects of cTBS over prefrontal cortex on CFFt. In a previous study, Carmel et al. [21] found a diffuse increase of activity detected by fMRI in prefrontal regions, i.e. bilateral middle frontal gyrus (BA 46), left medial frontal gyrus (BA 6) and right superior frontal gyrus (BA 6) during flickering visual stimuli perception. However, a similar pattern of diffuse prefrontal activity occurs in cognitive tasks which involve visual spatial attention and awareness of visual stimuli not related to temporal analysis, for example binocular rivalry and perception of bistable figures [78], [79]. Conversely, selective activation of left inferior parietal lobule occurs only during flicker perception. We believe therefore that the left IPL is involved in encoding conscious flicker perception, while bilateral prefrontal activity could have a more general role in orienting visual attention and visual awareness.

## Conclusion

Our study provides further insight into the physiological mechanisms of CFFt showing that left IPL plays a prominent role in CFFt encoding possibly according to a NMDA dependent-scalar model. Moreover, we demonstrated that CFFt is sensitive to manipulation by TMS plasticity inducing protocols. This new information may be useful for future studies in patients with movement disorders including dystonia [80]–[83] in which altered multimodal sensory temporal processing has been demonstrated.
